# ELISPOT Cell Analysis Assay: Searching for Extracellular Footprints

**DOI:** 10.3390/cells1010003

**Published:** 2011-09-21

**Authors:** Alexander E. Kalyuzhny

**Affiliations:** Neuroscience, UMN Twin Cities, 6-145 Jackson Hall, 321 Church St SE, Minneapolis, MN 55455, USA; E-Mail: kalyu001@umn.edu

I am honored to introduce the new journal *Cells*, which has been created to serve as a hub for disseminating new findings and discoveries in cell biology to researchers worldwide. *Cells* is an international, peer-reviewed, open-access journal on cell biology, molecular biology, and biophysics.

Much has been accomplished in molecular and cell biology in the past thirty years. We have unraveled the structure of many proteins, nucleic acids and lipids, and learned much about cell structures, including lysosomes, mitochondria, cytoskeletal elements, cell membranes, endoplasmic reticulum, and nuclei. Now, it is time to apply this knowledge to answering the age-old question: “How do cells work?” It appears there is a strong demand for a journal focused on various functional aspects of cell biology, including such fundamental ones as gametogenesis, embryonic development, tissue regeneration, tumorigenesis, and aging.

Although much progress has already been done, working through the further challenges that lay ahead should help us develop a better understanding of cellular structure and function. These challenges are great because in order to comprehend the magic of cellular machinery, we must analyze not only hundreds of intracellular biochemical events, but also unravel the relationships between them. How are normal cells triggered to turn into cancer cells? How can stem cells be used to heal wounds and replace damaged organs? Why do some cells have a longer life cycle than their adjacent neighbors of the same origin? Is cell aging reversible? For researchers working on these and other related projects, *Cells* will provide a perfect forum to share their findings with the rest of scientific community.

The scope of *Cells* can accommodate a wide variety of topics, including cell anatomy and physiology, organelles, cell adhesion and motility, intracellular signaling, apoptosis and aging, growth and differentiation, and techniques to study cell function, such as high-content screening, protein and gene arrays, immunocytochemistry, ELISA and ELISPOT. Manuscript submissions will be reviewed independently and anonymously to assure that only research papers of the highest standard are accepted for publication. Our goal is to turn *Cells* into a credible and high-ranking scientific journal so authors may feel proud to have their papers published in it. Our first issue will be dedicated to research on the ELISPOT cell analysis assay used either on its own or combined with other techniques, including immunocytochemistry, ELISA, *in situ* hybridization, flow cytometry, and other analytical techniques. To facilitate timely publication of novel findings, we aim for a rapid peer-review process for submitted papers to make the open access journal *Cells* a media of choice to researchers all over the world. 

On behalf of the Editorial Office, I wish to extend a warm welcome to *Cells*’ contributors. Let’s start this exciting journey together and make *Cells* a reliable scientific journal for many years to come.

